# Safety and Efficacy of Drug Holidays for Women with Sexual Dysfunction Induced by Selective Serotonin Reuptake Inhibitors (SSRIs) Other than Fluoxetine: An Open-Label Randomized Clinical Trial

**DOI:** 10.3390/brainsci13101397

**Published:** 2023-09-30

**Authors:** Elham Lalegani, Negin Eissazade, Mohammadreza Shalbafan, Razieh Salehian, Seyed Vahid Shariat, Sanaz Askari, Laura Orsolini, Shiva Soraya

**Affiliations:** 1 Mental Health Research Center, Psychosocial Health Research Institute (PHRI), Department of Psychiatry, School of Medicine, Iran University of Medical Sciences, Tehran 14496-14535, Iran; elhamlalegani90@gmail.com (E.L.); shalbafan.mr@iums.ac.ir (M.S.); salehian.razieh@gmail.com (R.S.); vahid.shariat@gmail.com (S.V.S.); drsanazaskari@gmail.com (S.A.); 2 Student Research Committee, School of Medicine, Iran University of Medical Sciences, Tehran 14496-14535, Iran; negin.eissazade@gmail.com; 3 Unit of Clinical Psychiatry, Department of Neurosciences/DIMSC, Polytechnic University of Marche, 60126 Ancona, Italy; l.orsolini@staff.univpm.it; 4 Research Center for Addiction and Risky Behaviors (ReCARB), Psychosocial Health Research Institute, Department of Psychiatry, School of Medicine, Iran University of Medical Sciences, Tehran 14496-14535, Iran

**Keywords:** arousal, desire, drug holidays, lubrication, orgasm, pain, selective serotonin reuptake inhibitors, sexual dysfunction, satisfaction

## Abstract

Selective serotonin reuptake inhibitors (SSRIs) are the cornerstone of psychopharmacology. However, they cause side effects such as sexual dysfunction, leading to the discontinuation of treatment. We aimed to investigate the efficacy and safety of drug holidays for women experiencing sexual dysfunction Induced by SSRIs other than fluoxetine. This study was an 8-week randomized, open-label, controlled trial including married women aged between 18 and 50 years who had experienced sexual dysfunction while undergoing treatment with SSRIs. The intervention group implemented drug holidays by not taking medications on Thursdays and Fridays, while the control group continued regular medication use. The female sexual function index (FSFI) and the 28-question general health questionnaire (GHQ-28) were administered to assess sexual function and mental health, respectively. A total of 50 participants completed the trial. The drug holidays’ group showed significant improvements in arousal (*p* < 0.001), desire (*p* = 0.001), orgasm (*p* < 0.001), satisfaction (*p* < 0.001), lubrication (*p* = 0.021), and overall sexual health (*p* < 0.001). The between-group difference of pain was significant (*p* < 0.001), despite no significant within-group change. Mental health improved in both groups, despite no significant between-group difference. No major adverse effects were reported. Drug holidays did not introduce immediate safety concerns or significant adverse effects during the timeframe of eight weeks, suggesting that it may be a safe and effective strategy for managing SSRI-induced sexual dysfunction in women, alongside improving mental health. Further research is needed to reach a definitive conclusion.

## 1. Introduction

Sexual dysfunction occurs in 15% to 80% of the patients undergoing treatment with selective serotonin reuptake inhibitors (SSRIs), often persisting long after discontinuation and significantly affecting sexual health and overall quality of life [1]. Notably, research suggests that female sex is a predictive factor for the severity of sexual dysfunction [2].

The most common forms of SSRI-induced sexual dysfunction are loss of libido, arousal disorders, genital anesthesia, dyspareunia, delayed orgasm, or anorgasmia [1]. SSRIs can impact various aspects of sexual function by increasing serotonin levels, decreasing dopamine and norepinephrine levels, blocking cholinergic and alpha-1 adrenergic receptors, elevating prolactin and free testosterone levels, and inhibiting nitric oxide synthetase [3,4]. Several strategies have been explored to address this issue, as discussed in a comprehensive 2013 Cochrane review: the ‘wait-and-see’ approach, behavior-changing techniques, psychotherapy, dose reduction, delaying the dose until sexual activity, switching to a different medication, adjuvant therapy, and drug holidays. However, due to a lack of diversity in clinical trials, no definitive conclusion can be drawn regarding the safety and efficacy of these strategies [5,6].

Drug holidays involves temporarily discontinuing or reducing the medication dosage to alleviate the associated side effects. Drug holidays has specifically been recommended for delayed orgasms and anorgasmia [5,6,7,8,9]. However, research in this area is limited, with only one 4-week clinical trial reporting significant improvement of sexual function, without any significant changes in depressive symptoms, except in those receiving fluoxetine, possibly due to fluoxetine’s long half-life [8,9]. With this knowledge gap in mind, our study aims to evaluate the safety and efficacy of drug holidays for women with sexual dysfunction induced by SSRIs other than fluoxetine.

## 2. Materials and Methods

### 2.1. Trial Setting and Design

This study was designed as an eight-week randomized, open-label, controlled trial, and it was carried out in the outpatient clinics of the Iran Psychiatric Hospital, the Rasoul-e-Akram General Hospital, and the Tehran Institute of Psychiatry (all affiliated with the Iran University of Medical Sciences in Tehran, Iran) from March 2022 to March 2023.

### 2.2. Participants

All the patients were assessed by a board-certified psychiatrist and their medical records were reviewed. Married (as sexual activity in Iran is generally culturally accepted within the context of marriage) sexually active women aged between 18 and 50 years of age who had experienced sexual dysfunction while undergoing treatment with SSRIs (other than fluoxetine) were included in the study. The patients were in the maintenance phase of their treatment, with a stable condition for the past two months and without any changes in their medication dosage. The exclusion criteria included the following: the use of fluoxetine (as drug holidays are reportedly ineffective due to fluoxetine’s long half-life); the use of medications other than SSRIs that are known to have sexual side effects (venlafaxin and duloxetin, tricyclic antidepressants, typical antipsychotics, risperidone, biperiden, and anticholinergics); and a poor medication adherence as reported by their psychiatrist [10].

The participants were randomly assigned to two groups using the block method (blocks of four): the drug holidays’ group and the control group.

### 2.3. Data Collection

Demographic data (age, education level, employment status, medication, and past psychiatric history) were collected. The participants completed the female sexual function index (FSFI) at baseline, at week four, and at the endpoint of the study [11,12,13]. The 28-question general health questionnaire (GHQ-28) was also completed at baseline and at the endpoint of the study to assess any changes in the overall mental health of the participants [14,15].

In addition, the signs and symptoms of adverse effects potentially associated with drug holidays were evaluated using a structured checklist at each visit [16].

### 2.4. Instruments

The FSFI is a six-dimensional self-report questionnaire designed to evaluate women’s sexual health. It consists of 19 questions that are categorized into six subscales: desire (two questions scoring from 1–5), arousal (four questions scoring from 0–5), lubrication (four questions scoring from 0–5), orgasm (three questions scoring from 0–5), satisfaction (three questions scoring from 0–5), and pain (three questions scoring from 0–5). The sum of each domain score is first multiplied by a domain factor ratio (0.6 for desire; 0.3 for arousal; 0.3 for lubrication; 0.4 for orgasm; 0.4 for satisfaction; and 0.4 for pain) and then subsequently summed to calculate the total FSFI score. The full-scale score ranges from two to thirty-six. Higher scores indicate a greater level of sexual health. The validity (*p* ≤ 0.001) and reliability (Cronbach’s alpha coefficient ≥ 0.7) of the Persian version of the FSFI scale were previously evaluated [11,12,13]. The GHQ-28 consists of four subscales: somatic symptoms, anxiety and insomnia, social dysfunction, and depression. Each question is scored from zero to three. Lower scores indicate better mental health. The Cronbach’s alpha, split-half coefficients, and test–retest reliability have been evaluated and reported as 0.9, 0.89, and 0.58, respectively, for the Persian version of the GHQ-28 [14,15].

### 2.5. Interventions

The participants in the drug holidays’ group were instructed not to take their medications on Thursdays and Fridays, corresponding to the weekends in Iran, when sexual intercourse is more likely to occur. This schedule was maintained for eight weeks. In contrast, participants in the control group were instructed to continue using their medications as prescribed without making any changes.

### 2.6. Sample Size and Statistical Analysis

The sample size for this study was calculated to be 50, with 25 participants in each group, based on a between-groups difference of four, a type I error of 5%, and a power of 80% [8]. The Mauchly’s test was employed to assess data sphericity, ensuring the validity of the repeated-measures ANOVA. In cases of sphericity violation, corrections were applied, using either the Greenhouse–Geisser or Huynh–Feldt epsilon adjustments, as appropriate. The repeated-measures ANOVA analysis was used to assess the effects of demographics and drug holidays over time on the FSFI scores. A post hoc pairwise comparison using the Bonferroni correction was conducted to compare scores between each pair of visits. The 28-GHQ scores were compared within each group by a paired *t*-test. A *p*-value of <0.05 was considered statistically significant. All the statistical analyses were performed using the Statistical Package for the Social Sciences (SPSS) software for Windows (Version 27, SPSS Inc., Chicago, IL, USA).

### 2.7. Ethics Approval and Consent to Participate

The trial was approved by the Ethics Committee of the Iran University of Medical Sciences Institutional Review Board (IR.IUMS.FMD.REC.1400.417) and was registered at the Iranian Registry of Clinical Trials (IRCT ID: IRCT20211027052886N1) prior to initiation. The trial was conducted in accordance with the principles outlined in the Declaration of Helsinki and its subsequent revisions. Written informed consent was obtained from all the participants, who were assured of the voluntary nature of their participation and their right to return to their usual treatment at any stage of the study.

## 3. Results

**Figure 1:**
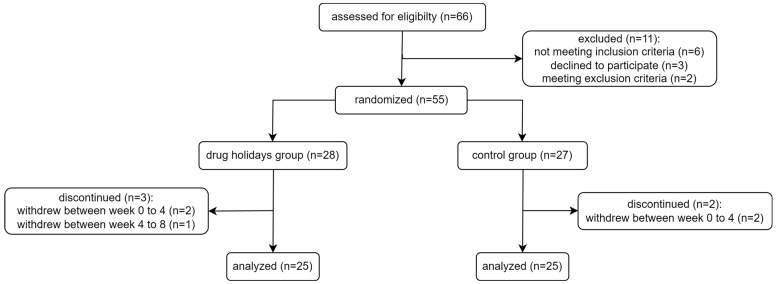
Flowchart of the participants of the study.

Out of the 66 female patients assessed for eligibility, 11 were excluded. A total of 55 participants were randomly assigned to either the drug holidays’ group (*N* = 28) or the control group (*N* = 27), and 50 patients successfully completed the trial (Figure 1). Demographic data of the participants are presented in Table 1.

At baseline, the mean scores of the FSFI, except for pain, exhibited significant differences between the two groups, indicating a greater severity of sexual dysfunction in the drug holidays’ group (Table 1).

### 3.1. Total

**Figure 2:**
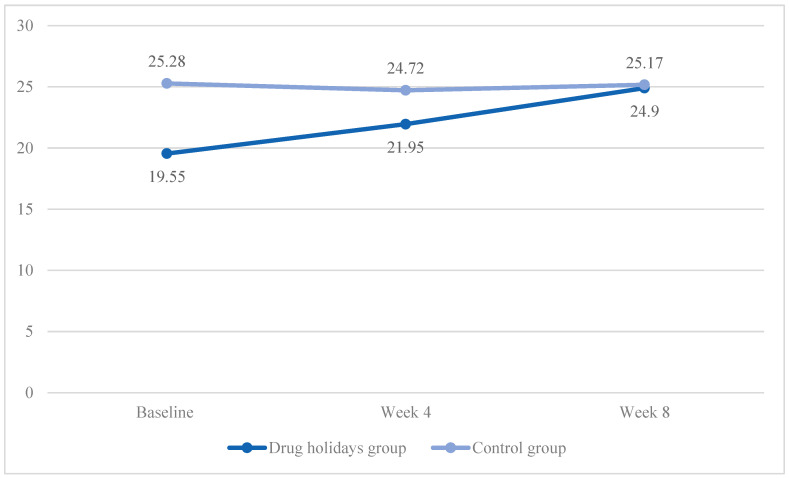
The FSFI total score changes of the participants during the course of the trial.

In the drug holidays’ group, the mean total score increased from 19.55 ± 5.17 at baseline to 24.9 ± 4.21 at the end of the trial, while, in the control group, it slightly decreased from 25.28 ± 4.62 to 25.17 ± 3.39 (Figure 2).

Sphericitywas violated, χ2 (2) = 10.485, *p* = 0.005. A significant Time X Treatment interaction effect was found between the groups: F(1.755, 84.262) = 16.691, *p* < 0.001.

The sphericity was met in the drug holidays’ group (χ2 (2) = 2.349, *p* = 0.31), and was violated in the control group(χ2 (2) = 12.6, *p* = 0.002). Furthermore, there was a significant mean score change within the drug holidays’ group (F(2, 48) = 27.665, *p* < 0.001) and a non-significant change within the control group (F(1.407, 33.763) = 0.470, *p* = 0.562).

### 3.2. Arousal

**Figure 3:**
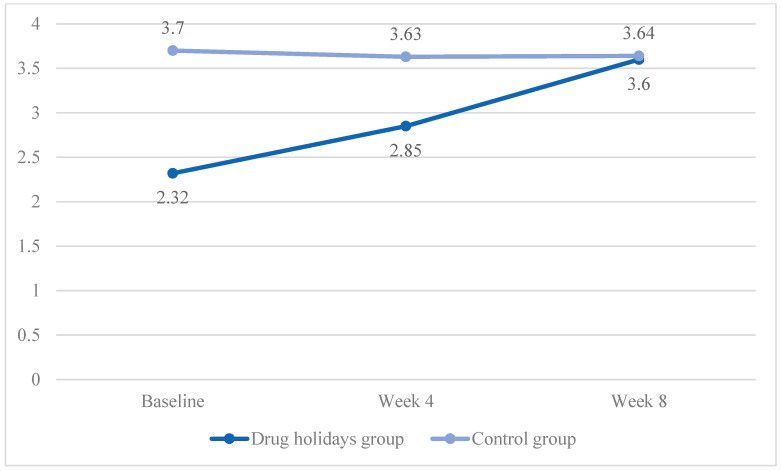
The FSFI arousal score changes of the participants during the course of the trial.

For the arousal subscale, the mean score increased from 2.32 ± 0.94 to 3.6 ± 0.81 in the drug holidays’ group and decreased from 3.70 ± 0.89 to 3.64 ± 0.74 in the control group (Figure 3).

The sphericity was met (χ2 (2) = 1.319, *p* = 0.517) and a significant Time X Treatment interaction effect was observed between the groups (F(2, 96) = 16.747, *p* < 0.001).

The sphericity was met in both the drug holidays’ group (χ2 (2) = 0.103, *p* = 0.95) and the control group (χ2 (2) = 4.91, *p* = 0.086). Additionally, there was a significant mean score change within the drug holidays’ group (F(2, 48) = 27.07, *p* < 0.001) and a non-significant change within the control group (F(1.407, 33.763) = 0.470, *p* = 0.562).

### 3.3. Desire

**Figure 4:**
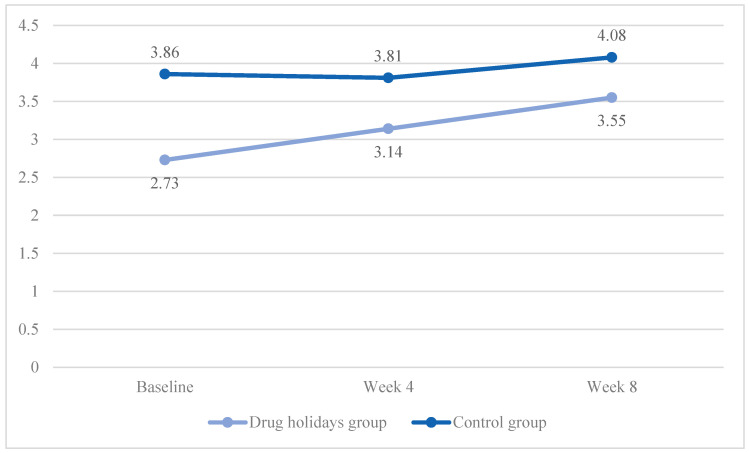
The FSFI desire score changes of the participants during the course of the trial.

The mean desire score increased from 2.73 ± 1.01 to 3.55 ± 0.66 in the drug holidays’ group and from 3.86 ± 0.96 to 4.08 ± 0.84 in the control group (Figure 4).

The sphericity was violated (χ2 (2) = 21.62, *p* < 0.001) and a significant Time X Treatment interaction effect was found between the groups: F(1.46, 70.13) = 4.29, *p* = 0.028.

The sphericity was violated in both the drug holidays’ group (χ2 (2) = 11.489, *p* = 0.003) and the control group (χ2 (2) = 9.805, *p* = 0.007). The mean score change was significant within the drug holidays’ group (F(1.436, 34.453) = 11.509, *p* = 0.001) and non-significant within the control group (F(1.485, 35.632) = 2.361, *p* = 0.122).

### 3.4. Orgasm

**Figure 5:**
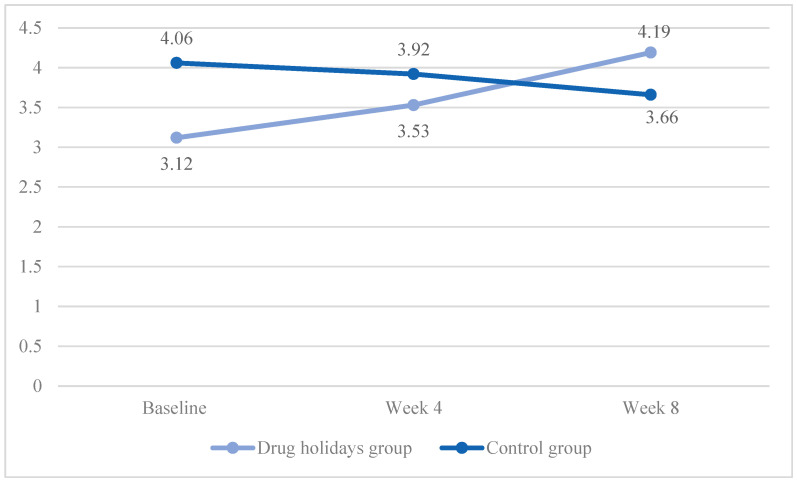
The FSFI orgasm score changes of the participants during the course of the trial.

In the drug holidays’ group, the mean orgasm score increased from 3.12 ± 0.92 to 4.19 ± 0.84, whereas, in the control group, it decreased from 4 ± 1.09 to 3.66 ± 1.03 (Figure 5).

As indicated by Mauchly’s test (χ2 (2) = 3.877, *p* = 0.007), the sphericity was violated, and a significant Time X Treatment interaction effect was found between the groups: F(2, 96) = 17.651, *p* < 0.001.

The sphericity was met in the drug holidays’ group (χ2 (2) = 5.667, *p* = 0.06), and was violated in the control group (χ2 (2) = 13.054, *p* = 0.001). The repeated-measures ANOVA indicated that the mean score change was significant within both the drug holidays’ group (F(1.746, 41.904) = 20.15, *p* < 0.001) and the control group (F(1.396, 39.396) = 20.150, *p* < 0.001).

### 3.5. Pain

**Figure 6:**
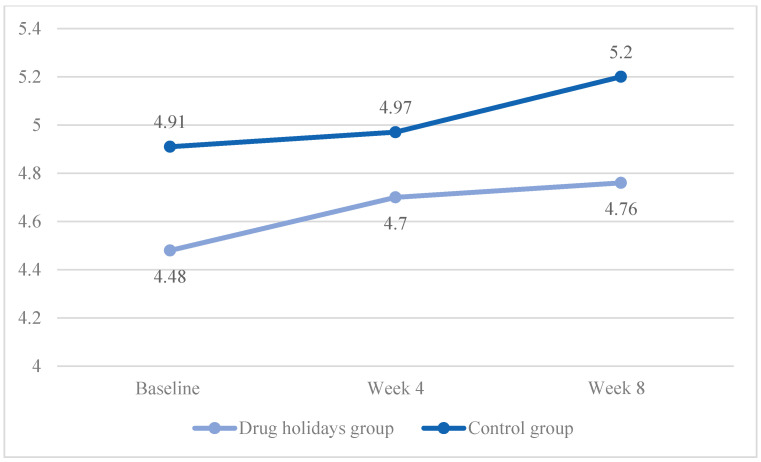
The FSFI pain score changes of the participants during the course of the trial.

The mean pain score increased from 4.48 ± 1.26 to 4.76 ± 0.92 in the drug holidays’ group and from 4.91 ± 1 to 5.2 ± 1.08 in the control group (Figure 6).

The sphericity was violated (χ2 (2) = 10.485, *p* = 0.005), and the Time X Treatment interaction effect was significant between the groups (F(1.755, 84.262) = 16.691, *p* < 0.001).

The sphericity was met in the drug holidays’ group (χ2 (2) = 4.354, *p* = 0.11), and was violated in the control group (χ2 (2) = 7.137, *p* = 0.028). The repeated-measures ANOVA indicated that the mean score change was non-significant within both the drug holidays’ group (F(1.823, 43.755) = 0.935, *p* = 0.393) and the control group (F(1.671, 40.109) = 1.485, *p* = 0.239).

### 3.6. Satisfaction

**Figure 7:**
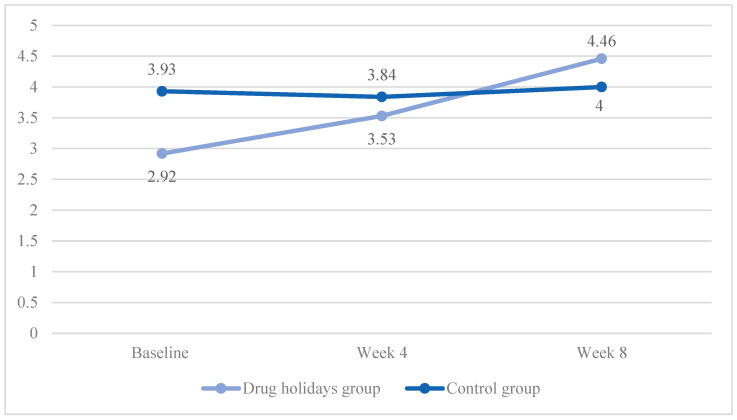
The FSFI satisfaction score changes of the participants during the course of the trial.

The mean satisfaction score increased from 2.99 ± 1.28 to 4.46 ± 1.23 in the drug holidays’ group and from 3.93 ± 1.11 to 4 ± 0.95 in the control group (Figure 7).

The sphericity was violated (χ2 (2) = 9.751, *p* = 0.008). A significant Time X Treatment interaction effect was found between the groups: F(1.775, 85.211) = 8.985, *p* < 0.001.

The sphericity was violated in the drug holidays’ group (χ2 (2) = 9.155, *p* = 0.01), and was met in the control group (χ2 (2) = 1.038, *p* = 0.6). The mean score change was significant among the drug holidays’ group (F(1.584, 38.026) = 15.115, *p* < 0.001) and non-significant among the control group (F(2, 48) = 0.347, *p* = 0.708).

### 3.7. Lubrication

**Figure 8:**
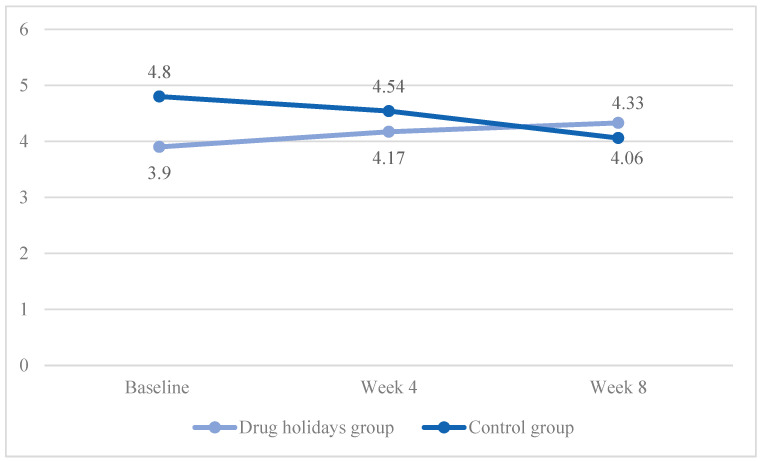
The FSFI lubrication score changes of the participants during the course of the trial.

The mean satisfaction score increased from 3.9 ± 1.09 to 4.33 ± 1.05 in the drug holidays’ group and decreased from 4.8 ± 1.05 to 4.54 ± 0.88 in the control group (Figure 8).

The sphericity was met (χ2 (2) = 2.484, *p* = 0.289), and a significant Time X Treatment interaction effect was found between the groups: F(2, 96) = 5.821, *p* = 0.004.

The sphericity was met in both the drug holidays’ group (χ2 (2) = 1.537, *p* = 0.46) and the control group (χ2 (2) = 1.537, *p* = 0.314). In addition, as revealed by the repeated-measure ANOVA analysis, the mean score change was significant within the drug holidays’ group (F(2, 48) = 4.196, *p* = 0.021) and non-significant within the control group (F(2, 48) = 1.984, *p* = 0.149).

The type of SSRI had a non-significant correlation with the above items (*p* > 0.05).

A post hoc analysis indicated that drug holidays significantly improved all the FSFI items, except pain. However, no significant change was observed within the control group, exceptfor the mean desire scores of the second and third visits (Table 2).

Furthermore, we conducted internal consistency reliability analyses using the Cronbach’s alpha and test–retest reliability analyses using the Pearson correlations. All were calculated to be between 0.7 and 0.8, confirming the reliability of our findings.

### 3.8. GHQ-28

The baseline GHQ-28 scores did not significantly differ between the two groups (*p* = 0.465). The mean GHQ-28 score decreased in both groups, from 42.2 ± 14.796 to 32.68 ± 12.612 in the drug holidays’ group and from 39.24 ± 13.63 to 33.96 ± 11.556 in the control group. The paired T-test analysis revealed that the decrease was significant within both the drug holidays’ group (*p* = 0.001) and the control group (*p* < 0.001). However, the difference between the two groups was not significant (*p* = 0.71).

### 3.9. Adverse Effects

The reported adverse effects in the drug holidays’ group were mild headache (N = 4, 4%), agitation (*N* = 2, 4%), and impaired concentration (*N* = 1, 8%).

## 4. Discussion

We found that, regardless of the type of SSRI (excluding fluoxetine), drug holidays significantly improved the arousal, desire, orgasm, satisfaction, lubrication, and the overall sexual health of our participants. The between-groups difference of pain was significant, despite no significant change within each group. In addition, mental health improved in both groups, despite no significant difference between the groups.

The baseline FSFI scores were significantly lower in the drug holidays’ group, indicating a more severe sexual dysfunction at baseline. However, compared to the control group, the sexual function of women in the drug holidays’ group significantly improved over time, suggesting that women with a more severe sexual dysfunction more likely achieve greater benefits from drug holidays.

Our findings align with the previous clinical trial conducted by Rothschild et al. (1995), which had a shorter follow-up period and a smaller sample size. Their 4-week trial investigated the effect of drug holidays on sexual dysfunction induced by sertraline, paroxetine, and fluoxetine in 14 men and 16 women. The patients were asked not to take their medications from Thursday morning until Sunday noon for four weeks. Among female patients treated with sertraline, 60% reported improved orgasmic function, and 40% experienced improved sexual satisfaction and libido. Similarly, among female patients treated with paroxetine, 40% reported improved orgasmic function, sexual satisfaction, and libido. No significant worsening of mental health status was observed in any of the patients. However, patients treated with fluoxetine did not report any improvements, possibly due to its long half-life [8]. Likewise, we found improvements in orgasm, satisfaction, and desire. Furthermore, a case report by Németh et al. (1996) reported the successful resolution of sexual dysfunction in a 22-year-old woman with major depressive disorder and obsessive-compulsive disorder who was experiencing anorgasmia while taking a daily dose of 300 mg of fluvoxamine. She was asked to decrease her dose to 100 mg on Fridays and Saturdays and then return to the regular dose on Sundays, and partial drug holidays completely resolved her sexual dysfunction. However, reducing the routine daily dose to 200 mg resulted in the recurrence of sexual dysfunction [9].

A 2013 Cochrane review was conducted on the management of SSRI-induced sexual dysfunction [4]. Thus far, various strategies have been studied, including the wait-and-see approach, psychotherapy, dose reduction, delayed dosing until after sexual activity, switching to a different medication, adjuvant therapy, and drug holidays. Each strategy has its advantages and limitations, and no conclusive evidence supports the superiority of one approach over another: In (1) the “wait-and-see” approach, a spontaneous resolution of sexual dysfunction may occur in 6–12% of the cases, with a marked or moderate improvement within 4–6 months. However, it may lead to non-adherence due to the significant required time. (2) Dose reduction may be considered if the underlying psychiatric disorder is well-controlled, as it carries the risk of symptom relapse. (3) Delaying the dose until after sexual activity may be effective for patients on antidepressants with short half-lives. However, planned sexual intercourse can cause stress and interfere with sexual performance and satisfaction. (4) Switching to a different medication is often preferred over adjunctive therapies as it improves adherence, reduces side effects and drug interactions, and decreases costs for the patient, e.g., mirtazapine and vortioxetine appear to have a lower risk of sexual dysfunction compared to SSRIs and serotonin–norepinephrine reuptake inhibitors (SNRIs). (5) Adjuvant therapy has been studied with various medications such as bupropion, buspirone, VML-670, granisetron, nefazodone, bethanechol, maca root, ginkgo biloba, and yohimbine. However, this approach requires careful follow-up and monitoring of drug interactions and side effects. Overall, so far, the most promising adjuvant therapy appeared to be adding bupropion at higher doses (150 mg twice daily) in women [4,5,17,18,19,20,21,22,23,24,25,26,27]. Lastly, (6) drug holidays provide a simple intervention that may enhance treatment adherence and reduce the likelihood of treatment discontinuation due to sexual dysfunction. Drug holidays have previously been recommended for delayed orgasms or anorgasmia. However, planned sexual activity during drug holidays may cause stress, potentially affecting sexual performance [4,5,19,28].

Our study supports the potential of drug holidays as an option for improving sexual health and maintaining treatment adherence in women with SSRI-induced sexual dysfunction. The decision to implement drug holidays should be made collaboratively through an informative and accurate discussion between the patient and their prescriber, taking into account the specific characteristics of the mental disorder, the severity of sexual dysfunction, and the potential risks and benefits of temporary medication discontinuation. However, further research is needed to investigate the optimal duration of drug holidays, their efficacy in different patient populations, and their long-term effects on sexual function and mental health.

### Limitations

Our study had several limitations, including an open-label design, a small sample size, a self-report bias, the exclusion of patients with comorbidities, and a limited generalizability. In addition, we did not evaluate the optimal duration of drug holidays. Different durations of medication discontinuation may have different effects on sexual function and mental health. The short follow-up period limited the detection of long-term effects of drug holidays. The variability in SSRIs, in combination with our small sample size, limited the breakdown of the mean score changes by medication. Also, the significant baseline differences of the FSFI scores affected our results. While our primary aim was to assess the effects of the intervention on sexual function, significant differences in baseline scores biased attributing the observed changes solely to the intervention. Moreover, we did not use a specific scale/questionnaire for the assessment of serotonin withdrawal symptoms, and we recommend that future studies develop and evaluate the validity and reliability of the Persian questionnaires/scales for the assessment of serotonin withdrawal symptoms. Additionally, future studies should aim for multicenter clinical trials with larger sample sizes, longer follow-up periods, and diverse populations (in terms of severity, clinical course, age ranges).

## 5. Conclusions

Regardless of the SSRI type (excluding fluoxetine), drug holidays significantly improved arousal, desire, orgasm, satisfaction, lubrication, and the overall sexual health of our participants. The between-group difference of pain was significant, despite no significant within-group change. Mental health improved in both groups, despite no significant between-group difference. Further research is needed to strengthen the body of evidence for the safety and efficacy of drug holidays in women with SSRI-induced sexual dysfunction.

## Figures and Tables

**Table 1:** Demographic data and the baseline mean (±SD) FSFI scores of the participants.

		Drug Holidays’ Group (N = 25)		Control Group (N = 25)		
		Mean (±SD)	Frequency (%)	Mean (±SD)	Frequency (%)	*p*-Value
Age (years)		35.52 ± 6.52		33.36 ± 8.40		
Education	High school diploma or lower		10 (40%)		13 (52%)	
	Higher education		15 (60%)		12 (48%)	
Employment	Employed		8 (32%)		5 (20%)	
	Unemployed		17 (68%)		20 (80%)	
Medication	Sertraline		13 (52%)		13 (52%)	
	Escitalopram		5 (20%)		6 (24%)	
	Paroxetine		4 (16%)		3 (12%)	
	Citalopram		-		1 (4%)	
	Fluvoxamine		3 (12%)		2 (8%)	
Previous psychiatric diagnosis	Depressive disorders		17 (68%)		11 (44%)	
	Anxiety disorders		4 (16%)		8 (32%)	
	Obsessive-compulsive and related disorders		4 (16%)		6 (24%)	
FSFI scores						
Arousal			2.32 ± 0.94		3.70 ± 0.89	<0.001 *
Desire			2.73 ± 1.01		3.55 ± 0.66	<0.001 *
Orgasm			3.12 ± 0.92		4 ± 1.09	0.001 *
Pain			4.48 ± 1.26		4.91 ± 1	0.294
Satisfaction			2.99 ± 1.28		3.93 ± 1.11	0.012 *
Lubrication			3.9 ± 1.09		4.8 ± 1.05	0.004 *
Total			19.55 ± 5.17		25.28 ± 4.62	<0.001 *

* *p*-values less than 0.05.

**Table 2:** The *p*-values of pairwise comparison of total and subscales’ score between visits in the drug holidays’ group and the control group.

		Arousal	Desire	Orgasm	Pain	Satisfaction	Lubrication	Total
Drug holidays’ group								
Baseline	Week 4	0.021 *	0.079 *	0.135	0.885	0.018 *	0.136	0.003 *
Week 4	Week 8	0.001 *	0.004 *	<0.001 *	1	0.012 *	0.999	0.001 *
Baseline	Week 8	<0.001 *	0.002 *	<0.001 *	0.847	<0.001 *	0.041 *	<0.001 *
Control group								
Baseline	Week 4	1.00	1.00	0.963	1	1.00	0.269	0.896
Week 4	Week 8	1.00	0.039 *	0.356	0.43	1.00	1.00	1.00
Baseline	Week 8	1.00	0.585	0.307	0.585	1.00	0.425	1.00

* Significant difference between two visits.

## Data Availability

The datasets used and analyzed during the current study are available from the corresponding author on reasonable request.

## Abbreviations

SSRIs	Selective serotonin reuptake inhibitors
FSFI	Female sexual function index
GHQ-28	28-question general health questionnaire
